# EPR-based uncertainty validation of the calculated external doses for population exposed in the urals region

**DOI:** 10.1093/rpd/ncac238

**Published:** 2023-09-18

**Authors:** E A Shishkina, M O Degteva, B A Napier

**Affiliations:** Biophys. Lab., Urals Research Center for Radiation Medicine, 68A Vorovsky Str., 454124, Chelyabinsk, Russia; Department of Radiation Biology, Chelyabinsk State University, 129 Bratiev Kashirinykh Str., 454001, Chelyabinsk, Russia; Biophys. Lab., Urals Research Center for Radiation Medicine, 68A Vorovsky Str., 454124, Chelyabinsk, Russia; Pacific Northwest National Laboratory, 902 Battelle Boulevard, Richland, Washington, 99354, USA

## Abstract

Tooth enamel Electron Paramagnetic Resonance (EPR) spectroscopy was used as a method for external dosimetry in the territories contaminated in the 1950s by PA ‘Mayak’ (Urals region) to validate the mean dose estimates predicted by the Techa River Dosimetry System (TRDS). The purpose of this study is to validate the uncertainties of TRDS doses. Ninety percent confidence intervals (90% confidence interval, CI) of dose estimated with both methods were compared for 220 people. All data were grouped according to the width of 90%CI, viz.: (1) 90%CI of EPR-based dose ≤  90%CI of TRDS prediction (38 cases); (2) 90%CI of EPR-based dose >  90%CI of TRDS prediction (182 cases). About 91% of 90%CIs overlap. In group 1, 100% cases overlap. In group 2, 80% of the cases were non-contradictive (the calculated 90%CI is completely within the measured one). Interval comparison of doses predicted retrospectively and estimated based on individual measurements are non-contradictory and demonstrate a good agreement.

## Introduction

Electron Paramagnetic Resonance (EPR) measurements of teeth are widely used in retrospective dosimetry to validate theoretically derived external doses^(1, 2)^. Particularly, EPR was used as a method for external dosimetry in the territories contaminated in the 1950s by Production Association ‘Mayak’ (Urals region) to validate the external dose estimates predicted by the Techa River Dosimetry System (TRDS) using group-average approach^(3, 4)^. TRDS is the basis for epidemiological studies in the Urals region. For example, risk analyses based on the deterministic version of TRDS (TRDS-2016D) have established associations between radiation doses and the occurrence of solid cancer and leukaemia for the Techa River Cohort^(5)^. However, dose uncertainty can result in biased risk estimates and underestimation of the risk uncertainty. Therefore, the current version of TRDS includes stochastic modelling (TRDS-2016MC) to predict both expected doses (point estimates) and the corresponding uncertainties (interval estimates) for exposed individuals^(6, 7)^ for the correction of confidence intervals in excess relative risk models^(8)^.

The TRDS-2016MC approach is to use Monte Carlo replications of the basic model (TRDS-2016D) with uncertain input parameters. It should be noted that stochastic modelling includes assumptions and simplifications, and the interval dose estimates need validation too. The analysis of the calculations with the previous version of TRDS shows an underestimation of the uncertainties of external exposure doses^(9)^. The purpose of the study is the validation of external dose uncertainties predicted by the current version of TRDS-2016MC using EPR tooth dosimetry.

The validation of TRDS uncertainty can be done using interval comparison of modelled and EPR-based dose reconstructions for individuals^(9)^. The individual multiple dose realisations in TRDS-2016MC were compared with individual EPR-based doses taking into account the uncertainty. The method of interval comparison strongly depends on the uncertainties of the validated and validating doses. Based on the analysis of the uncertainties of parallel TRDS-2016MC and EPR-based dose reconstruction, approaches to how to provide the interval comparison to validate both individual TRDS-2016MC doses and corresponding uncertainties were proposed and applied.

## Materials and methods

### Sample description

Four hundred ninety-four EPR measurements of teeth from 221 people were suitable for the analyses^(9)^. Donors were born in 1913–43. Most of them (173 persons) lived permanently in the Techa riverside settlements over the period from 1950 through 1952. Other persons lived either part-time at the Techa riverside, migrated between settlements with different levels of contamination, or migrated to the most contaminated area of the East Urals Radioactive Trace. External TRDS doses were calculated using age-dependent air-to-enamel dose conversion factors^(10)^ and with due account of an additional contribution of ^137^Cs circulating in the surrounding soft tissues^(3)^. TRDS dose predictions for the selected individuals vary from 2 to 600 mGy (median is 50 mGy; the 25–75% range corresponds to 24–120 mGy). Standard dose uncertainty in terms of coefficient of variation (CV) is in the range of 30–120%. Dose uncertainty depends on availability of household location data for an individual, exposure associated with the age-dependent time spent at the shoreline, as well as period of residence in the contaminated territories. The individuals with doses < 50 mGy were mostly short-term residents and/or were too young to visit the river on their own (according to observations and surveys old people spent 3 times less time on the contaminated river bank than teenagers). The uncertainty typical of such doses is on average 60%. For doses in the ranges of 50–100, 100–300 and >300 mGy the mean uncertainties are 70, 81 and 95%, respectively. More than half of individuals in the sample are permanent residents of the highly contaminated villages, which were evacuated in 1954–56. For such people, the main factor of both dose formation and dose uncertainty is age during the contact period. The tendency of uncertainty increase with dose is a result of increase of variability in life patterns for different age groups. These dose uncertainties are the sample characteristic and are not typical of the whole exposed population.

Description of the EPR methods, data harmonisation, subtraction of the contributions of natural background and bone-seeking ^90^Sr as well as treatment of non-detects and averaging of individual doses of different precisions in different teeth was provided in Shishkina *et al*.^(3)^. Because of internal and background dose subtraction, 36% of EPR doses are negative. EPR-derived doses in the sample under study are from −79 up to 2300 mGy (median is 17 mGy; 75% are < 160 mGy). Uncertainties of EPR doses were discussed in detail in Shishkina *et al*.^(9)^. Uncertainties of measurement-based doses have a reverse tendency of dose-dependence: CV ~ 350% for doses < 50 mGy; for doses in the ranges of 50–100 mGy, 100–300 mGy, 300–500 mGy and >500 mGy the mean relative standard uncertainties are 200, 70, 40, and <30%, respectively. In contrast to the modelling uncertainty (which includes the limitation of our knowledge), the relative measurement uncertainty (CV) decreases with an increase in the measurand.

The width of 90% confidence interval, CI of EPR-based doses was higher than that of TRDS-2016MC for 83% of individuals. It was mainly typical of the individuals with expected TRDS doses < 120 mGy. Only 17% of individuals have 90%CI of EPR-based doses lower than that of TRDS-2016MC.

### Problem statement and general approaches

**Figure 1:**
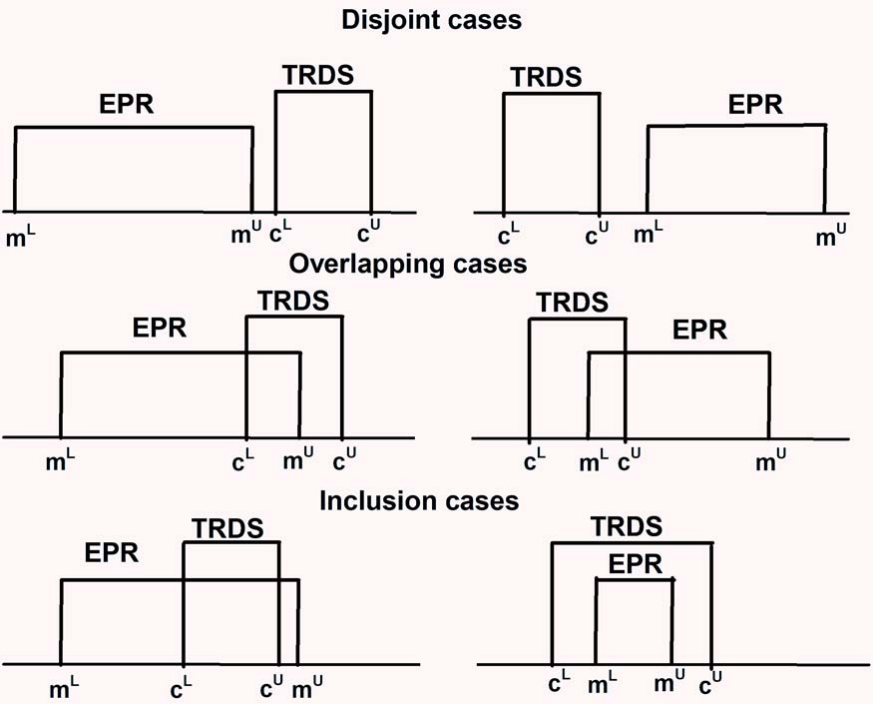
Interval relations.

Comparisons of the uncertain data were performed based on analysis of 90% confidence interval (90%CI) matching. For EPR-based dose estimates the 90%CI is presented as *EPR* = [m^L^, m^U^]; for TRDS-216MC the 90%CI is *TRDS* = [c^L^,c^U^]. Possible interval relations are shown in Figure 1.

EPR uncertainty ranges can include negative numbers, which does not make sense in terms of the ‘true dose value’. Therefore, if m^L^ < 0, *EPR* = [0, m^U^]. To compare intervals, both qualitative and quantitative indices can be used. Qualitative indexes are useful for consistency testing. In the framework of the current study, two estimates were qualified as non-contradictory if the condition expressed in Equation (1) is satisfied (shown as overlapping or inclusion in Figure 1).


(1) \begin{equation*} EPR\cap TRDS>0 \end{equation*}

This is a very lenient criterion, since the interval boundaries may just touch or slightly overlap. A stricter estimator could be formulated depending on the uncertainty of validating and validated doses. If the validating dose interval is wider than that of validated one, then one can expect that the calculations should completely fall within the TRDS interval (the left-side inclusion case in Figure 1). Equation (2) formulates a more rigorous consistency criterion for very uncertain EPR-based doses.


(2) \begin{equation*} EPR> TRDS,\left( EPR\cap TRDS\right)/ TRDS=1 \end{equation*}

If *EPR ≤ TRDS*, then the greater is the overlapping fraction, *f*, of *EPR* (Equation (3)) the more reliable is the calculation uncertainty:


(3) \begin{equation*} EPR\le TRDS,f=\left( EPR\cap TRDS\right)/ EPR \end{equation*}

The mean *f* of a selection is a non-parametric estimator of consistency, reflecting the mean *TRDS* coverage of *EPR*.

For the precise EPR-based data (*EPR ≤ TRDS*), we can estimate a quantitative index (*P_c_*). *P_c_* reflects the coverage probability assuming normality for measurement-based doses and lognormality for TRDS-2016MC doses^(7)^ (Equation (4)).


(4) \begin{equation*} {P}_c=\frac{P_{D_{EPR}}\left( EPR\cap TRDS\right)\times{P}_{D_{TRDS-2016 MC}}\left( EPR\cap TRDS\right)}{P_{D_{EPR}}(EPR)\times{P}_{D_{TRDS-2016 MC}}(TRDS)} \end{equation*}

where ${P}_{D_{EPR}}( EPR\cap TRDS)$ is the probability of EPR dose, which takes the value in the interval *EPR ∩ TRDS*;

${P}_{D_{TRDS-2016 MC}}( EPR\cap TRDS)$ is the probability of TRDS-2016MC dose prediction, which takes the value in the interval *EPR ∩ TRDS*;

${P}_{D_{EPR}}(EPR)$ is the probability of EPR-based dose which takes the value in interval *EPR*;

${P}_{D_{TRDS-2016 MC}}(TRDS)=0.9$ is the probability of TRDS-based dose which takes the value in 90%CI *TRDS*.

Coverage probability is the probability of EPR and TRDS-based doses are simultaneous hitting within the coverage region EPR ∩ TRDS. A qualitative index of Pc > 0.5 can be assumed to be an indicator of a good agreement of TRDS-predicted CI and EPR-based interval estimates (the probability of a simultaneous hitting is >50%).

## Results and discussion

All data were subdivided into two groups as follows: *EPR* ***≤*** *TRDS* and *EPR > TRDS*. Table 1 presents the description of the groups.

**Table 1:** Description of data groups classified according to the widths of confidence intervals.

Parameter	*EPR ≤ TRDS*	*EPR > TRDS*
Sample size	38	183
Mean EPR-based dose (min–max), mGy	120 (−100–980)	170 (−200–2400)
Mean TRDS-based dose (min–max), mGy	240 (30–450)	60 (2–600)
Mean m^L^—mean m^U^, mGy	60–370	74–450
Mean c^L^—mean c^U^, mGy	50–660	19–132

### The lenient criterion of consistency

All the data of the *EPR ≤ TRDS* group meet the lenient criterion of consistency (Equation (1)) corresponding to either the overlapping and inclusion cases in Figure 1. In the second group, ~90% of data match the criterion. In total, 91% of measurement and calculation pairs could be assumed as non-contradictory. The remaining 9% can be interpreted as discrepancies between calculations and measurements, which may be a result of some untraced individual history of contacts with the contaminated territory.

### The stricter estimators of consistency

In group of *EPR > TRDS*, the cases of *EPR* inclusion into *TRDS* (Equation (2) and left-side illustration of inclusion in Figure 1) were assumed as non-contradictive in terms of both dose and uncertainty prediction by TRDS-2016MC. Eighty percent of measurement and calculation pairs fulfil the criterion. The remaining 20% includes all the cases of non-compliance with the lenient criterion. Additional 10% of inconsistent data do not demonstrate any obvious pattern that allows us to conclude about the reason for the unsatisfactory comparison. This is 19 cases of residents of different age who resided in different settlements for which the predicted point dose estimates are in the range of 13–600 mGy; EPR-derived doses are in the range from non-detects up to 2 Gy. Nevertheless, point estimates for these 19 cases are highly correlated (*r* = 0.869, *p* < 0.000002) and this means that we most likely underestimate the uncertainty of TRDS doses.

**Figure 2:**
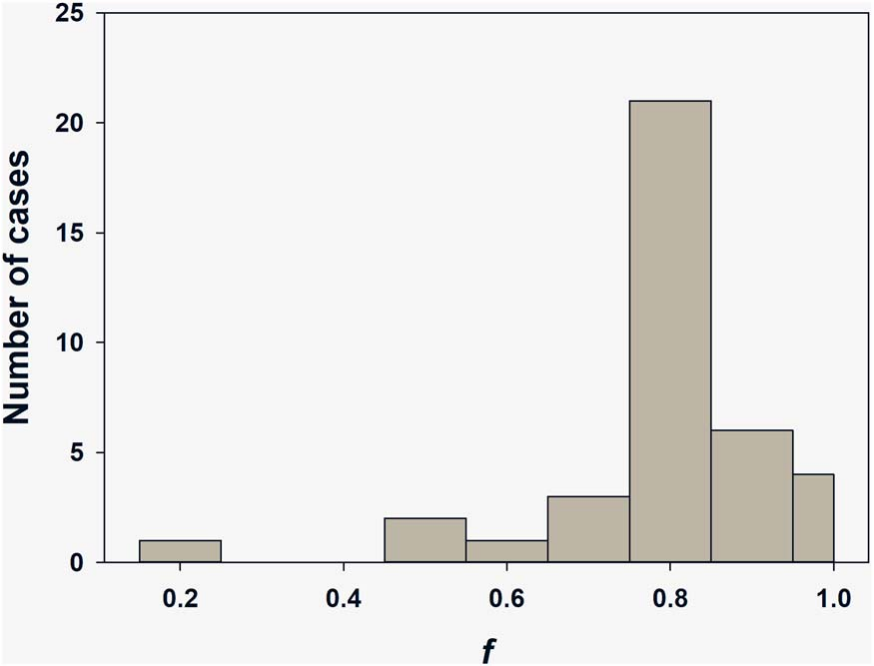
Distribution of individual *f*-values in group with *EPR ≤ TRDS.*

In group of *EPR* ***≤*** *TRDS*, the estimator of consistency, *f*, (Equation (3)) is a measure of the *EPR ∩ TRDS* of *EPR* (right-side illustration of both overlapping and inclusion in Figure 1). Figure 2 illustrates the distribution of individual *f-*values.

From Figure 2, the *f*-value is lower than 0.5 for one case only; *f*-values are > 0.8 for 80% cases. Mean *f*-value is equal to 84%. EPR doses in these group includes non-detects for which a point estimates are unavailable for an individual dose comparison. However, based on dose uncertainty we can estimate the upper border of 90%CI of EPR-based dose. The truncated (from zero dose to the upper border) EPR interval is used for the comparison. This is the advantage of the interval approach.

### The criterion of agreement

**Figure 3:**
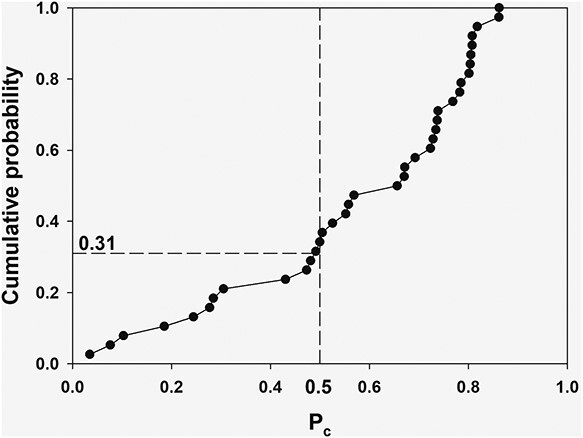
Cumulative distribution of coverage probability, *P_c_*, in group with *EPR ≤ TRDS.*

For the EPR-based data assumed to be precise (*EPR ≤ TRDS*) the coverage probability, *P_c_*, was calculated according to Equation (4). Figure 3 presents the cumulative distribution of *P_c_*.

The TRDS-2016MC-predicted 90%CI is in good agreement with experimental data if *P*_c_ > 0.5. This is true for ~70% of the data. Such individual agreement is quite good for retrospective dosimetry in terms of mean dose prediction. However, ~30% of the *TRDS* intervals perhaps are somewhat underestimated. This serves as an indicator of TRDS-2016MC uncertainty underestimation. It should be noted that the underestimation is not dramatic. Uncertainties of TRDS-2016MC predictions indicated to be underestimated are, on average, ~100% in terms of coefficient of variation. TRDS-2016MC uncertainties of well-matching data are, on average, ~115%.

## Conclusion

Interval comparison of doses predicted retrospectively and estimated based on individual measurements are in general non-contradictory. Only 20% of calculated doses are inconsistent with measurement results: 9% can be assumed as disagreement due to untraced individual contacts with a high contaminated territory; the remaining 11% can be associated with underestimation of TRDS dose uncertainties. Seventy percent of cases demonstrate the good agreement between measurements and calculations. Current estimates of external dose uncertainties of TRDS-2016MC prediction are quite reasonable. Further efforts will be aimed at eliminating the existing slight underestimation.
